# Utilizing the ultrasensitive *Schistosoma* up-converting phosphor lateral flow circulating anodic antigen (UCP-LF CAA) assay for sample pooling-strategies

**DOI:** 10.1186/s40249-017-0368-1

**Published:** 2017-11-01

**Authors:** Paul L. A. M. Corstjens, Pytsje T. Hoekstra, Claudia J. de Dood, Govert J. van Dam

**Affiliations:** 1 Department of Molecular Cell Biology, Leiden University Medical Center, P.O. Box 9600, 2300 RC Leiden, The Netherlands; 2 Department of Parasitology, Leiden University Medical Center, P.O. Box 9600, 2300 RC Leiden, The Netherlands

**Keywords:** Schistosomiasis, Diagnostics, Sample pooling, Mass drug administration, Circulating anodic antigen, Lateral flow, Strip test, Up-converting reporter particle, UCP-LF CAA

## Abstract

**Background:**

Methodological applications of the high sensitivity genus-specific *Schistosoma* CAA strip test, allowing detection of single worm active infections (ultimate sensitivity), are discussed for efficient utilization in sample pooling strategies. Besides relevant cost reduction, pooling of samples rather than individual testing can provide valuable data for large scale mapping, surveillance, and monitoring.

**Method:**

The laboratory-based CAA strip test utilizes luminescent quantitative up-converting phosphor (UCP) reporter particles and a rapid user-friendly lateral flow (LF) assay format. The test includes a sample preparation step that permits virtually unlimited sample concentration with urine, reaching ultimate sensitivity (single worm detection) at 100% specificity. This facilitates testing large urine pools from many individuals with minimal loss of sensitivity and specificity. The test determines the average CAA level of the individuals in the pool thus indicating overall worm burden and prevalence. When requiring test results at the individual level, smaller pools need to be analysed with the pool-size based on expected prevalence or when unknown, on the average CAA level of a larger group; CAA negative pools do not require individual test results and thus reduce the number of tests.

**Results:**

Straightforward pooling strategies indicate that at sub-population level the CAA strip test is an efficient assay for general mapping, identification of hotspots, determination of stratified infection levels, and accurate monitoring of mass drug administrations (MDA). At the individual level, the number of tests can be reduced i.e. in low endemic settings as the pool size can be increased as opposed to prevalence decrease.

**Conclusions:**

At the sub-population level, average CAA concentrations determined in urine pools can be an appropriate measure indicating worm burden. Pooling strategies allowing this type of large scale testing are feasible with the various CAA strip test formats and do not affect sensitivity and specificity. It allows cost efficient stratified testing and monitoring of worm burden at the sub-population level, ideally for large-scale surveillance generating hard data for performance of MDA programs and strategic planning when moving towards transmission-stop and elimination.

## Background

Schistosomiasis is a chronic and debilitating disease, most prevalent in Africa but also in other parts of the tropics and subtropics [1]. The disease is caused by an infection with the helminth *Schistosoma*. Generally, different species infecting humans are geographically localized depending on the presence of species-specific freshwater snail vectors. The main human-targeting schistosome species of concern are *S. haematobium* and *S. mansoni* in Africa, *S. japonicum* and *S. mekongi* in Asia [2, 3]. Control strategies, such as preventive chemotherapy, mass drug administration, MDA, with the anti-schistosomal drug praziquantel (PZQ) treating school children, are being used in high *Schistosoma* endemic settings to reduce the burden of infection, prevent development of severe morbidity, and decrease prevalence [4–6]. This approach is usually accompanied by improved access to clean water, sanitation and hygiene (WASH; [7]), as well as snail control and community education. Accurate mapping of the disease and monitoring efficiency of different approaches to reduce morbidity and to break transmission still mostly depend on determination of eggs in urine (urine filtration technique [8]; for urogenital schistosomiasis: *S. haematobium*) or stool (Kato-Katz technique [9]; for intestinal schistosomiasis: *S. mansoni*, *S. japonicum* and *S. mekongi*) by microscopy. These assays have a high specificity, but results are often variable and lack sensitivity in areas with low worm burden and near-elimination, transmission-stop, settings [10–13]. Moreover, in cases where drug treatment only temporarily suppresses egg production infections are easily missed; in this context, recovery of affected adult worms or maturation of young worms that are less susceptible to the drug [14, 15] as well as rapid reinfections, are known concerns [16]. As deposition of the eggs in either stool or urine depends upon the *Schistosoma* species, in areas of mixed infections or when dealing with unknown species, both stool and urine need to be examined making mapping of low endemic areas a laborious and time-consuming enterprise. Therefore there have been repeated calls for highly accurate genus specific diagnostics with all species detectable in the same non-invasively collectable biological matrix and preferably deployable in the field [12, 17–19].

For a number of years, a well validated laboratory-based test with excellent accuracy using urine has been available for detection of active infections of all different *Schistosoma* species [20, 21]. This test, an Up-Converting Phosphor Lateral Flow (UCP-LF) based assay [22], detects the *Schistosoma* Circulating Anodic Antigen (CAA) which is a glycosaminoglycan-like carbohydrate with a unique structure [23]. The CAA test should not be confused with the commercially available, field-friendly, Point-Of-Care Circulating Cathodic Antigen (POC-CCA) test which detects a different *Schistosoma* antigen [24]. The POC-CCA detects a ‘cathodic’ antigen [25] in urine whereas the UCP-LF CAA test detects an ‘anodic’ antigen in blood and urine and can be used in any other body fluid. The indication ‘cathodic’ and ‘anodic’ relates to their migration behaviour in an electric field [26]. Both antigens are regurgitated by *Schistosoma* worms in the blood stream of the human circulation [27, 28] and end up in urine; the rapid clearing mechanisms have not been determined in full detail. The POC-CCA test was developed as a rapid, field-friendly assay for detection of *S. mansoni* infections in urine and is widely used for mapping and monitoring as an excellent alternative to the Kato-Katz (KK) stool analysis [29, 30]. POC-CCA is a qualitative assay allowing visual interpretation of the test results. CCA structurally has some homology with the class of Lewis X antigens which may limit the specificity of the POC-CCA. In contrast, the UCP-LF CAA test was developed as a sensitive quantitative test for detection of all *Schistosoma* species, and current test formats allow detection of a single worm pair, or single sex worm infections [31–33]. The UCP-LF CAA test includes a sample preparation step and a UCP-LF strip reader to analyse the test result. The pooling applications described here are only applicable for CAA, as its unique biochemical structure allows almost unlimited concentration from urine samples.

The sample preparation step comprises an acidic treatment of the urine sample with trichloroacetic acid (TCA), leaving CAA in a protein-free supernatant after centrifugation. The resulting clear solution can be concentrated to increase sensitivity using dedicated molecular weight cut-off filtration devices. When using urine as a clinical sample, high concentration factors can be achieved [33]. Presence of CAA in urine is indicative of an active ongoing infection [34]. Measurements correlate qualitatively with eggs detected in stool for intestinal schistosomiasis (*S. mansoni* and *S. japonicum*; [34–36] or eggs detected in urine samples for urogenital schistosomiasis (*S. haematobium*, [27]). Importantly, CAA concentrations can be used as an accurate and direct measure of worm burden [35]. The correlation may vary somewhat between different species, variability in urine production or factors such as differences in immune status of the host, chronicity and intensity of the infection, or reduced metabolic activity i.e. worms recovering from drug treatment. Considering that urine can be obtained non-invasively and in large volume, it is a very practical clinical sample also in terms of sample pooling. An additional advantage is that CAA in urine is stable for several days or weeks at ambient temperature, thus not requiring a cold-chain when testing is performed away from the sample collection site, e.g. in a centralized laboratory. The test has been validated in several scientific studies, worldwide under different conditions [31, 37–39]. Based on experimental data, the assumption is that analysis of the concentrate of 2 mL of urine with the UCP-LF CAA assay with an analytical sensitivity < 0.1 pg/ml will identify the majority of active infections.

In this manuscript large scale urine pooling is considered and potential applications are discussed that can be relevant to mapping, hot spot detection, mass drug administration (MDA), efficiency of drug treatment, re-emergence of infection, monitoring transmission stop, surveillance, and reduction of the number of diagnostic tests when requiring identification of infected individuals in low prevalence settings.

## Methods

The current manuscript describes a methodological approach for using different formats of the UCP-LF CAA test in ‘diagnostic’ applications with pooled urine samples. This methods section describes the relevant and practical details required to perform the test. Source information regarding required materials and equipment is given at the end of the result section. Actual practical data as determined from a random available sample set before and after drug treatment serves the purpose of demonstrating validity of accurate testing urine pools with the UCP-LF CAA test.

### Procedure of the *Schistosoma* genus specific CAA test

#### General description of the UCP-LF CAA strip test

**Fig. 1:**
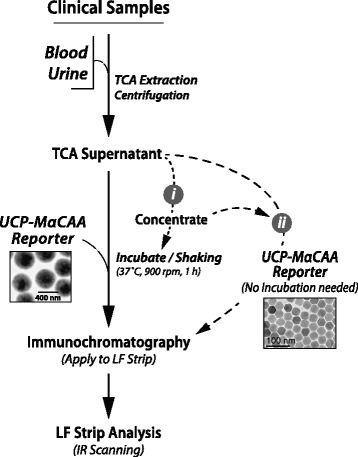
Schematic of the UCP-LF CAA assay. Clinical samples as blood (whole blood, serum or plasma, eluted dry blood stains) and urine are extracted with TCA. (i) After centrifugation, sensitivity can be boosted by concentration of the TCA-sup using ultra-centrifugal filtration devices, e.g. applicable for sample pooling purposes. TCA-sup is incubated with the MαCAA-UCP reporter conjugate (400 nm YOS particles); (ii) incubation can be omitted when using nano-sized (< 100 nm)YF particles. Following incubation the mixture is applied to a LF strip with CAA-capture line. LF strips are analysed using e.g. battery operated portable readers for single strip interrogation (UCP-Quant) or the benchtop multistrip readers (UPCON), provided with an IR excitation source and appropriate software

The assay can be performed with all body fluids including dried blood samples [40] and is optimized for samples extracted with trichloroacetic acid (TCA) [22]. The TCA extraction separates antigen-antibody immune complexes and precipitates protein material while leaving CAA and possibly other carbohydrate structures in solution. Following extraction, centrifugation-clarified material (TCA-sup) is mixed and incubated (1 h, 37 °C, 900 rpm) with the luminescent UCP reporter coated with mouse monoclonal anti-CAA antibodies (UCP-MαCAA). This pre-flow incubation improves sensitivity when using ‘large’ (400 nm) YOS reporter particles [41], but may be omitted upon implementation of novel smaller (< 100 nm) NaYF particles [42]. The amount of sample input can be increased by concentrating TCA-sup using Amicon filtration devices [33]. UCP-MαCAA/TCA-sup mixtures are examined by immunochromatography on LF strips with a test line comprised of mouse monoclonal anti-CAA antibodies [22]. Test results are analysed quantitatively by scanning LF strips for UCP reporter signals with dedicated strip readers. A summary of the different steps is shown in Fig. 1. A more detailed description of the different formats of the assay can be found in previous publications [20, 33, 37]. The description below is focused on testing urine samples, in particular the TCA extraction and sequential concentration steps to increase sensitivity as these form the basis of the pooling applications.

#### TCA extraction of urine samples

Extraction of the clinical samples is performed at a final concentration of 2% (*w*/*v*) TCA. Protocols optimized for serum analysis require mixing of equal volumes of serum and 4% (*w*/*v*) TCA [22]. The same protocol is applicable for urine, but extraction of a low viscosity sample allows mixing with a higher percentage TCA solution. Up to 40% (w/v) TCA can be used, although care should be taken as it may lead to some gas formation when patients are burdened with urolithiasis. After thorough mixing with TCA, samples are left at ambient temperature for 5 min for efficient denaturation of proteins after which the mixture is clarified by centrifugation; the carbohydrate structure CAA remains in the soluble fraction, TCA-sup. As an alternative for centrifugation, a combination of disposable glass- and paper-filters could be applied for removal of the TCA-precipitate.

#### Concentration of urine TCA-sup

To improve sensitivity TCA-sup can be concentrated using 10 kDa molecular weight cut-off membranes, available as Amicon ultra-centrifugal filtration devices up to 15 ml or larger cell pressure-based filtration devices (> 50 ml). The maximum level of concentration is depending on the final viscosity of the concentrated TCA-sup. The TCA-sup from urine samples does allow rigorous concentration; e.g. using the 15 ml Amicon centrifugal device a 1400-fold concentration can be obtained (see below UCP-LF CAA assay formats: UCAA1400).

### UCP-LF CAA assay formats for urine testing

The standard UCP-LF CAA test for urine is performed with 10 μl urine extracted with an equal volume of 4% (*w*/*v*) TCA. This assay is referred to as UCAA10: ‘U’ indicating that the sample is urine, ‘CAA’ indicating the target and ‘10’ indicating the amount of urine in μl analysed on the LF strip. This terminology has been introduced previously [20] and is used in follow-up publications [31–33, 38, 43, 44]. The standard format does not involve concentration of the TCA-sup, while all the other formats described in this manuscript (Tab. 1) do include a concentration step of the TCA-sup. The final TCA concentration in the supernatant is always 2% (*w*/*v*), but the TCA concentration in the solution used for extraction can differ (Tab. 1). The amount of sample is maximized for the concentration devices used. In case clinical sample volume is limited, lower amounts can be used with appropriate adaptation of the TCA solution such that extraction is always performed at 2% (*w*/*v*) final concentration [43].

**Table 1:** UCP-LF CAA assay formats for urine analysis

*Assay* *format*	*Analyzed* *volume (μl)*	*TCA extraction solution* *volume (μl) / % (w/v)*	*Amicon (centrifugal)* *concentration device*	*QC threshold* ^1^ *singlet test (pg/ml)*
UCAA10	10 μl	10 μl / 4%	none	10
UCAA250	250 μl	250 μl / 4%	0.5 ml Filter Tube	1
UCAA2000	2 000 μl	2 000 μl / 4%	4 ml Filter Tube	0.1
UCAA14000	14 000 μl	1 000 μl / 30%^2^	15 ml Filter Tube^3^	0.03
UCAA40000	40 000 μl	10 000 μl / 10%^2^	50 ml Cell^3^	0.01

^1^Quality control threshold (CAA concentration) for single test with wet reagents [20]

^2^Utilizing relevantly higher TCA concentration than the 4% (*w*/*v*) applied in the standard formats

^3^The 15 ml filter tube and 50 ml cell concentration is a two-step procedure requiring an additional 0.5 ml filter tube to concentrate down to 20 μl

In this manuscript sample volumes of 10, 250, 2 000 and 14 000 μl are considered depending on the required analytical sensitivity level. Generally, in high endemicity settings the UCAA10 assay can be applied to provide a good indication of prevalence of infection by individual diagnosis (using a 10 μl urine sample). The UCAA250 assay is valuable to follow the efficiency drug administration at the individual level when monitoring individuals from high endemic settings. The UCAA2000 assay, when applied for individual diagnosis using 2 000 μl urine sample, allows detection of low infection levels and detects the majority of single worm infections. For various pooling applications the UCAA250, −2000, −14000 and −40 000 may be used; the number and volume of samples to be pooled will depend on the objective. The objective of pooling can relate to obtaining information at the population level (infected individual not identified) or methods to reduce costs involved with the actual diagnostic test.

### UCP-LF CAA test materials and specific equipment

The UCP reporter particles, mouse monoclonal anti-CAA antibody (MαCAA), and conjugation conditions are identical to the materials used during initial development of the UCP-LF CAA assay [22]. Lateral flow (LF) strip materials as described previously [45] were obtained via Kenosha C.V., (Amstelveen, the Netherlands). Test (MαCAA) and Flow Control (goat anti-mouse, GαM) capture lines were applied to the nitrocellulose part of the LF strip utilizing an automatic TLC Sampler 4 (CAMAG, BCON Instruments, Sint Annaland, the Netherlands). LF strips were produced with the same composition of Test and Flow Control lines as described in a recent study demonstrating the use of Amicon ultra centrifugal filter devices to concentrate urine samples thereby enhancing analytical sensitivity [33]. The Amicon filtration devices were obtained from Merck-Millipore. UCP-LF strips were analysed with the UPCON ELISA plate reader, applicable for reading 20 LF strips at a time (Labrox Oy, Turku, Finland) but can also be analysed using other readers with UCP capability, e.g. the custom adapted portable ESEQuant *LFR* reader (QIAGEN Lake Constance GmbH, Stockach, Germany) [45].

### Clinical samples

A panel of 162 urines was kindly provided by Dr. Gilles Riveau (Biomedical Research Center Espoir pour la Santé, St Louis, Senegal). These samples were collected from 81 infected children (7–12 years of age) living in the hyper-endemic area of Senegal River Basin and were part of a larger study conducted in the area (https://clinicaltrials.gov/ct2/show/NCT00870649?term=bilhvax&rank=2); urine was collected from each individual before and 6 months after drug administration. Importantly, samples were tested with the UCP-LF CAA test as an example to demonstrate that average CAA levels measured in pools matched the average calculated from individual samples. With the available information it is not possible to draw conclusions regarding reinfection frequency and potential effects of vaccination included in the clinical trial.

## Results

### UCP-LF CAA and urine pooling for stratified population-based mapping purposes

Mapping, monitoring and evaluation strategies mainly deal with prevalence, intensity of disease, and examination of cure after PZQ treatment. These programmes need high quality data on infection levels and worm burdens, preferably in a quantitative manner [17]. Information resulting from detection of the directly worm-derived CAA in relevantly larger groups of people living in endemic areas will have great advantages over a more granulated testing schedule [46]. The fact that *Schistosoma* transmission is highly focal (‘hot-spots’) adds to the significance of this approach [47].

Below we describe a practical pooling approach that involves the use of the UCAA2000 assay. This specific assay allows pooling of up to 200 urine samples of 10 μL each. The TCA-sup of this urine pool is concentrated 200-fold such that it can be analysed on a UCP-LF CAA test strip, similar to the UCAA10 assay. Within this urine sample pool a single individual with a positive UCAA10 assay result would already provoke a ‘CAA positive’ test result of the pool. Similarly, a total of 25, 1400 or even 4000 samples can be pooled when using respectively the UCAA250, −14000 or −40000 assay formats (Tab. 2). The result will be the CAA concentration of the pool as an average per person in pg/ml, and can be interpreted as a rough measure of the worm burden in the tested population. The number of diagnostic tests will be relatively low with respect to the number of individuals included in the test. Pool sizes of 25 (UCAA250) and 200 (UCAA2000) seem to be the most appropriate when considering school and village mapping. The system is fully flexible, allowing increase of the urine sample volume, with appropriate decrease of the number of samples pooled when sensitivities below the UCAA10 level are required. Moreover, the pooling approach also is convenient for stratified testing, e.g. stratification based on age, sex, occupation, distance to the lake, etc.

**Table 2:** Relation between pool size, individual sensitivity (sample volume) and concentration device

*Assay format*	*Concentration device*	*Number of pooled samples*
UCAA10	None	1 × 10 μl
UCAA250	0.5 ml Filter Tube	25 × 10 μl; 1 × 250 μl
UCAA2000	4 ml Filter Tube	200 × 10 μl; 8 × 250 μl; 1 × 2 000 μl
UCAA14000	15 ml Filter Tube	1 400 × 10 μl; 56 × 250 μl; 7 × 2 000 μl
UCAA40000	50 ml Cell	4 000 × 10 μl; 160 × 250 μl; 20 × 2 000 μl

**Fig. 2:**
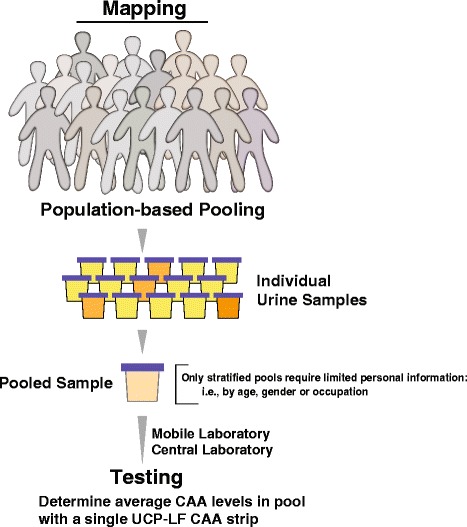
Pooling for population-based mapping. Smaller or larger urine collections can be pooled to determine with a single UCP-LF CAA test the average CAA level in the pool. No personal data or records are needed when analysing a complete or representative group of a village. For specific stratified testing, some personal information/record to conclude that individuals fit the targeted group is needed

Pooling approaches as depicted in Fig. 2 can be applied in high and low endemic areas. When considering mapping of low endemic areas, in particular transmission stop or elimination settings, large pool sizes can be used. A concentration device is needed, as the average CAA level in the pool is likely below the UCAA10 level. When mapping medium to high endemic settings, large concentration factors may not be required. In hyper-endemic settings such as the River Basin of Senegal, concentration of the TCA-sup prepared from the urine pool may require dilution rather than concentration.

### UCP-LF CAA and urine pooling for monitoring drug treatment efficiency and re-infection

With the population-based mapping approach as indicated above, a measure for infection incidence could be the average CAA concentration in the test group. This clearly is not the same as the prevalence of infection (percentage of infected individuals), but can be considered as a valuable alternative. The occurrence of individual high intensity infections may disturb the overall result and separate analysis of smaller pools or a small set of individual samples may still be desired to confirm actual local prevalences [48]. However, more elaborate pooling strategies that have been established for other diseases and diagnostic testing that involve testing of overlapping pools may be adopted to resolve these issues. At this point, proof-of-principle is needed to demonstrate empirically the practicality of using population-based CAA levels for mapping purposes.

**Fig. 3:**
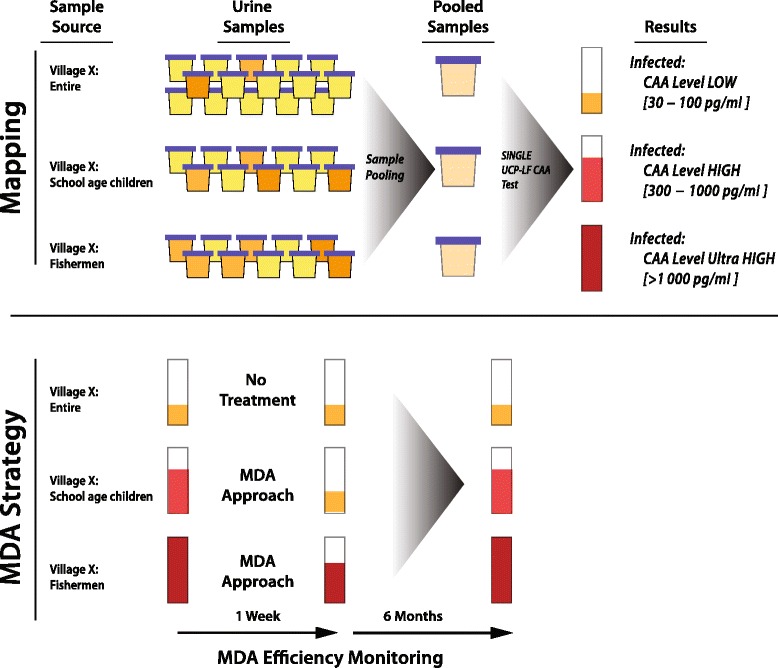
Average CAA levels to guide and monitor MDA decision and protocols. Hypothetical example illustrating the use of urine pools for MDA decision, monitoring drug treatment efficiency and re-infection. The indicated time scales for testing after drug treatment could be considered but are theoretical and may differ depending on the *Schistosoma* species and endemicity level. Monitoring the efficiency of the MDA drug treatment (after 2 days to a week) shows that the average CAA level is reduced, which is directly related to worm burden and worm activity. In this example it does also indicate that a second round might be favourable. Monitoring after 6 months shows that the old situation has recovered due to re-infection

Additionally, population-based CAA mapping results could be very useful to guide decisions in current MDA approaches (Fig.3). It provides a convenient method for monitoring the efficiency of protocols applied for PZQ treatment in a group and consequently allows optimization of protocols (e.g. amount and frequency of drug administration). Comparison of the CAA concentration in pools collected immediately before and shortly after drug administration will be an accurate measure for PZQ efficiency. This approach has major advantages compared to monitoring based on egg count methods, not only because of the poor sensitivity of egg count methods, but eggs can still be found in urine or stool weeks after worms have died and therefore do not allow monitoring shortly after drug treatment. Moreover, young worms (schistosomula) and worms that have temporarily ceased egg production as a consequence of PZQ treatment will not be noticed. UCP-LF CAA testing will still detect these worms, albeit that CAA production will be dependent on the age and health status of the worm. Following population-based CAA levels over time in longitudinal studies after PZQ treatment may also provide valuable information regarding recovery of PZQ affected worms and re-infection.

### Pooling and individual case identification

**Fig. 4:**
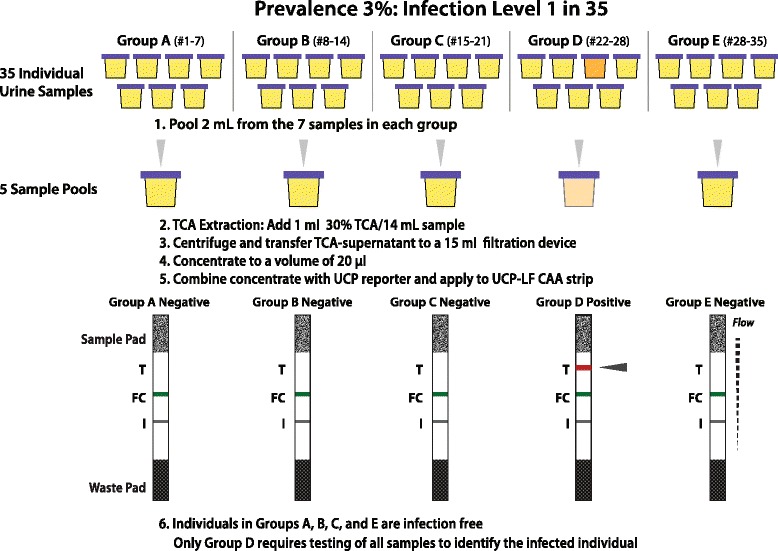
Schematic, illustrating the steps involved in pooling while maintaining ultimate sensitivity. Pools, prepared from individual samples of each 2 000 μl, that generate a ‘CAA-negative’ test result with the UCP-LF CAA assay do not require further analysis; all included samples/individuals can be regarded as UCAA2000 negatives, not carrying a schistosome infection. Test line (T), Flow Control line (FC) and Index line (I) are indicated: T, the binding site of the UCP^MαCAA^-CAA complex; FC the binding site for all UCP^MαCAA^ material not retained at T; I, non-essential index line with a fixed amount of label that can be used for normalization of the strip reader

Pooling strategies as described in the previous paragraphs, target population-based test results, which don’t need identification of the infected individual, and as such do not require full sensitivity. When identification of infected individuals is the goal (e.g. in more focused test-and-treat strategies), full sensitivity must always be maintained even when using pooling strategies. The pooling approach described in this section is based on reducing the number of diagnostic tests to identify individuals with a ‘CAA-positive’ test result (a CAA value above the threshold, indicating infection). Considering the UCAA2000 as the ultimate test for demonstrating infection, the urine sample volume of each individual in the pool must be equal to the volume used when testing a single individual with the UCAA2000 test. Pooling strategies benefit maximally when targeting test conditions that result in the largest number of ‘CAA-negative’ urine pools as samples from negative pools do not need individual analysis. Fig. 4 illustrates the steps involved as an example utilizing the UCAA14000 format to combine 7 urine sample of 2000 μl each. After TCA extraction (utilizing 1 ml 30% (*w*/*v*)), the clear TCA-sup is concentrated to 20 μl using the 15 ml Amicon filtration devices. This is a two-step process as the TCA-sup is first concentrated to 100 μl and then transferred to a 0.5 ml Amicon centrifugal device for final concentration to 20 μl. The two-step concentration process allows pooling of 7, 14, 21, 28 or 35 urine samples, 2 ml each, to be tested on a single strip; the optimal pool size is dependent on the expected prevalence. The example illustrated in Fig. 4 shows the identification of the 7-sample pool containing the only *Schistosoma* infected person in a group of 35 (prevalence of infection ~3%). This type of testing will be most valuable in low endemic settings as that likely implies a low prevalence of infection, hence a large number of negative pools. Individuals from ‘CAA-negative’ pools that contained 2 ml urine sample from each included person are presumed not to carry *Schistosoma* worms. A larger number of negative pools results in a  lower number of individual tests required to identify the infected persons. In Tab. 3 the number of pools and individuals that require re-testing is indicated in relation to the prevalence of infection. Individuals in ‘CAA-positive’ pools require retesting; secondary pooling strategies can also be applied for those pools, but these strategies are not further discussed here. The above examples are straightforward, indicating the potential use of the UCP-LF CAA test in pooling strategies focusing on individual case identification especially in low prevalence settings. Obviously, the lower the prevalence the lower the number of individuals that require an individual test. The pooling approach however is highly flexible and dependent on the objective pursued. While elimination requires ultimate sensitivity, a less stringent detection level may be sufficient to achieve transmission interruption. Moreover, the in Tab. 3 presented calculations do assume a uniform distribution of the ‘CAA-positive’ individuals, actually the most unfavourable situation as it maximizes the number of ‘CAA-positive’ pools. In reality, positive samples will cluster and increase the number of negative pools and increase the number of individuals not requiring an individual test result.

**Table 3:** Pooling strategy maintaining ultimate (UCAA2000) sensitivity

	Number of pools	*Number of positive individuals*		
Individuals	pool size = 35	Prevalence 5%	Prevalence 1%	Prevalence 0.2%
35	1	2	1	1
70	2	4	1	1
140	4	7	2	1
280	8	14	3	1
560	16	28	6	2
1 120	32	56	12	3
	Number of pools	*Number of positive pools*		
Individuals	pool size = 35	Prevalence 5%	Prevalence 1%	Prevalence 0.2%
35	1	1 (100%)	1 (100%)	1 (100%)
70	2	2 (100%)	1 (50%)	1 (50%)
140	4	4 (100%)	2 (50%)	1 (25%)
280	8	8 (100%)	3 (37.5%)	1 (12.5%)
560	16	16 (100%)	6 (37.5%)	2 (12.5%)
1 120	32	32 (100%)	12 (37.5%)	3 (9.4%)
	Number of pools	*Number of individuals not requiring an individual test*		
Individuals	pool size = 35	Prevalence 5%	Prevalence 1%	Prevalence 0.2%
35	1	0	0	0
70	2	0	35	35
140	4	0	70	105
280	8	0	175	245
560	16	0	350	490
1 120	32	0	700	1 015

Relation between prevalence and number of negative pools and samples not requiring an individual test results. At low prevalence the number of negative pools increases, hence less individuals require an individual result implying relevant reduction of the number of tests. Calculations are based on equal distribution of the ‘CAA-positive’ individuals, the least favourable condition leading to the highest number of individual tests. In real pooling applications, the number of negative pools expectedly will be higher than shown the above example due to non-random distribution of the ‘CAA-positive’ individuals

### A practical example to demonstrate sample pooling

**Fig. 5:**
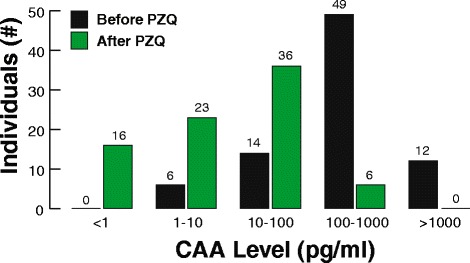
Frequency histogram of CAA concentration in 81 children before and after PZQ treatment

Urine samples from 81 children living in a hyper-endemic area in Senegal (River Basin) all carrying a *Schistosoma* infection were analysed with the UCAA10 and UCAA250 assay to diagnose *Schistosoma* infection status (Tab. 4). Out of 81, 72 children tested ‘CAA-positive’ with the standard UCAA10 assay (QC threshold of 10 pg/ml). When using the UCAA250 assay (QC threshold of 1 pg/ml) all 81 children tested positive. A distribution of the CAA concentration is shown in Fig. 5. All children received the same drug treatment (PZQ) based on their estimated weight. After 6 months, new urine samples were collected and individually analysed with the UCAA250 assay format. Qualitative analysis indicated that out of 81, 16 children returned a ‘CAA-negative’ result. Quantitative analysis showed that the average level of CAA dropped from 519 to 33 pg/ml, indicating the efficacy of the PZQ treatment. With the UCAA test the direct effect of the PZQ treatment on the viability of the schistosomes can be measured in urine pools already shortly (a few days) after treatment. With longer post-treatment periods as the 6 month used here, maturation of unaffected young worms, reinfection with new worms, as well as recovery of affected but not killed worms may also have resulted in detectable CAA levels.

**Table 4:** Pooling experiment with urine samples from River Basin children

*Study site*	River Basin, Senegal
*Included*	81 children (7–12 years of age) with high susceptibility of being infected
*Prevalence before PZQ*	89% (72 children) based on individual UCAA10 diagnosis
	100% (81 children) based on individual UCAA250 diagnosis
*CAA level* ^*1*^	519 pg/ml
*Prevalence after PZQ*	43.2% (35 children) based on individual UCAA10 diagnosis
	86.4% (65 children) based on individual UCAA250 diagnosis
*CAA level* ^*1*^	33 pg/ml
*POOLING*	Pooling of samples collected after PZQ treatment
*Pool composition*	Nine pools, each comprising an equal volume of 9 children –‘9 sample pool’
	One pool comprising an equal volume of all 81 samples – ‘81 sample pool’
*Applied test*	Pools were analysed with the UCAA250 format
*CAA level 9-sample pool* ^*1*^	47, 84, 29, 57, 60, 36, 22, 40, 33 pg/ml – average of nine ‘9 sample pools’ 45 pg/ml
*CAA level 81-sample pool* ^*1*^	40 pg/ml

^1^All concentrations indicate the average amount of CAA per individual, either of the whole selection (*n* = 81) or for the number of individuals in a specific pool

To evaluate the correlation of CAA levels determined in pools to those determined by individual testing, urine samples collected after PZQ treatment were combined into nine pools each with 9 individual urine samples (‘9- sample pool’; Tab. 4) as well as a single pool comprised of all 81 samples (‘81-sample pool’; Tab. 4). TCA-sups from the pools were analysed with the UCAA250 assay format and results compared to the levels obtained with the average of the appropriate individual samples. The average CAA level of all 81 individual samples (33 pg/ml) correlated well with the average nine ‘9-sample pools’ (45 pg/ml) and the level determined for the ‘81-sample pool’ (40 pg/ml). The correlation of the CAA concentrations using the nine ‘9-sample pools’ with the respective average CAA concentrations calculated from the individually tested samples was 0.86 (Pearson’s rho, *P* < 0.01) (Tab. 4).

The above experiment is an example of how a urine pooling strategy can be applied and demonstrates that average CAA levels determined from pools correlate well with the average of individually determined levels. CAA levels determined in pools can provide straightforward data for population-based mapping purposes and serve as input for computer modelling. The use of urine pools to monitor overall effectiveness of PZQ treatment in MDA approaches is also straightforward. However, more specific data is needed before CAA-pooling protocols can be adequately adapted for MDA- concerning decisions. An in depth analysis of previous studies that have applied the UCP-LF CAA assay format could provide some insight on the dependency of pooling approaches of prevalence level and infection grade. In high endemic regions with high worm burdens and consequently high CAA levels, decisions regarding MDA may be accepted quicker as involved individuals are probably well aware of *Schistosoma* related morbidity and may therefore comply easier to drug treatment without requesting an individual diagnostic test. Rationally, acceptance of treatment without an individual positive diagnostic test will decline with decreasing prevalence. In such a situation stratified pooling (e.g. based on profession or age) to identify smaller high risk groups appears useful.

## Discussion

The UCP-LF CAA assay for serum and urine has been reported in many scientific studies and has demonstrated superior diagnostic performance allowing accurate determination of true prevalences with close to 100% specificity and sensitivity [31, 33]. The main factors accountable for its high (analytical) sensitivity are: i) the high affinity of the anti-CAA mouse monoclonal IgG antibody; ii) the unique structure and stability of the CAA biomolecule and its solubility in trichloroacetic acid allowing concentration, and; iii) the ultra-sensitive background-free UCP reporter particle technology. Ongoing efforts are focussing on adapting the current user-friendly assay format, which requires some basic laboratory equipment to allow rapid point-of-care applications as needed for test-and-treat approaches in low endemicity and post MDA settings. Here we report and discuss another aspect, the potential of the current UCP-LF CAA assay format with pooled urine samples to obtain population-based test results and to identify individual low grade infections.

Several studies have indicated the applicability of the ultra-sensitive UCP-LF CAA test platform to identify individuals with low *Schistosoma* infection levels using non-invasive urine samples. In the majority of the cases, the measured CAA concentration can be used as a reliable guide for worm burden and correlates to egg production and subsequently to transmission. Although it must be recognized that CAA levels cannot yet be directly related to numbers of worms per infected individual, hence they do not always reveal the actual parasitology and clinical situation at the individual level, the presence of CAA always indicates an ongoing/active infection. Renal clearance of CAA from human circulation is only a matter of hours to few days [36, 49]. Taking into account differences between individuals and worm species, CAA concentrations before and after PZQ administration, longitudinal studies can provide relevant information about the short- and long-term drug efficacy, as well as other mechanism leading to continuing worm infections. Two formats of the UCP-LF CAA test, the SCAA500 (for serum) and the UCAA2000 assay (for urine), have been applied in several studies [31–33, 38, 44] and may detect a single worm pair. Although desired, the assays (yet) don’t support actual field POC testing mainly because of the requirement of centrifuges in the sample preparation step. The current format, developed with the goal to make the assay as sensitive as possible, also includes the use of centrifugal filtration devices. These devices are convenient for use in the laboratory, but are costly and difficult to implement in real field applications. Ongoing efforts are now focussing on less expensive and more field-friendly alternatives for concentration and/or extraction. Meanwhile, and in particular for urine testing, pooling strategies using the current formats to reduce the number of tests and associated costs are definitely feasible.

Although the theoretical examples of potential pooling approaches described here are unpretentious representations of the real situation, they indicate suitability of the UCP-LF CAA test for applications involving pooling approaches in high as well as low endemic settings (including the transmission-stop and elimination areas). For stratified mapping, monitoring of MDA efficiency and re-infection, the pooling approach can provide a rapid quantitative value on which actions can be based. Clearly, studies are needed to investigate optimal pool-sizes in relation to expected endemic levels, as well as the effect of hot-spots, incidental super-excretors, etc. In high endemic settings this type of pooling approach is straightforward and probably well acceptable by the ‘diagnosed community’ even if no individual test results are provided. However, additional studies demonstrating that average CAA levels determined from pools correlate with the average CAA levels determined from the individuals are needed; the data presented here for the Senegal selection successfully demonstrated pooling competence. When moving towards low endemicity, transmission stop and even elimination settings, it is obvious that the amount of each individual sample (urine volume) added to the pool needs to be increased with evident increase on the total volume of the urine pool. Where in high endemicity settings individual urine volumes of 10 μl (or even less) can be sufficient, in elimination settings individual samples of 2000 μl may be required to identify a single individual with a low infection level (single worm) in the pool. Pooling in such cases will still be efficient and save diagnostic costs, but it needs to be considered that in these situations identification of the infected individual is likely a requirement for individual drug treatment.

## Conclusions

We propose that at the population level, average CAA concentrations in urine can be an appropriate measure to indicate worm burden. Urine pooling strategies allowing this type of large scale testing are feasible with the various UCP-LF CAA test formats. Overall mapping, stratified testing of high risk groups (e.g. based on age, profession or habitat), and identification of hot spots, can be done easily with only a limited number of diagnostic tests. The determined population-based CAA level can provide essential data and impact treatment considerations. Moreover, as drug efficacy can be monitored through a decrease in CAA levels measured before and shortly (within days/weeks) after PZQ treatment, rapid identification of areas (becoming) insensitive to the applied MDA approach (including development of potential drug resistance) is practicably feasible. Finally, urine pooling protocols while maintaining the highest sensitivity (required in low endemicity setting as transmission-stop and elimination sites) and at the same time allowing identification of the infected individual(s) can be designed and relevantly reduce the number of diagnostic tests and related costs.

## Abbreviations

CAA: Circulating anodic antigen
CCA: Circulating cathodic antigen
GαM: Polyclonal anti-mouse antibody raised in goat
LF: Lateral flow
MDA: Mass drug administration
MαCAA: Monoclonal anti-CAA antibody raised in mouse
UCAA#: UCP-LF CAA test performed on # μL urine
UCP: Up-converting phosphor
